# Supplementary material for: Backward compatibility of whole genome sequencing data with MLVA typing using a new MLVAtype shiny application for Vibrio cholerae
Source: PLoS One. 2019 Dec 11;14(12):e0225848. doi: 10.1371/journal.pone.0225848 (PMC6905556; doi:10.1371/journal.pone.0225848)
Supplement: Attachment — Submitted filename: Response_to_reviewer.doc [file pone.0225848.s001.doc]

**We thank the Editor and the Reviewer for their careful reading of our manuscript. In revising our paper, we carefully followed the editor’s direction, and replied to the point by point questions of the reviewer.**

**We are confident that the answers provided and the corresponding modifications in the revised version will now meet the Editor and the Reviewer’s expectations.**

**We thank you in advance for your editorial work.**

**The authors**

Reviewer #1: Ambroise et al describe a software (MLVAtype) which allows to deduce MLVA genotypes from assembled Whole Genome Sequence (WGS) data. The input is a draft assembly, the size of the k-mer used for the assembly and the sequence of the repeated motif. MLVAtype will search for the largest occurrence of repeated motifs. Then MLVAtype will compare the length of this largest occurrence and will consider it as a valid VNTR allele if it is smaller than the k-mer value used for the assembly.
To evaluate MLVAtype, the authors used 19 V. cholera isolates and an MLVA assay comprising five loci with a 6 bp (3 loci), 7 bp or 9 bp repeat unit. They sequenced the PCR amplification products by Sanger sequencing to constitute the reference data. They then compare in vitro and WGS-derived MLVA typing data with the reference data.
Nine isolates were typed in vitro using the Agilent 2100 Bioanalyzer whereas ten isolates were typed in vitro using the higher resolution capillary electrophoresis system GenScan. 300 bp long WGS data was produced for the nine isolates versus 150 bp for the ten isolates. The 300 bp long reads allow to deduce a full and correct MLVA genotype whereas the 150 bp reads provide partial data. 90% of in vitro MLVA profiles are incorrect.

There are a number of issues associated with the software design, in vitro typing, report organization.

Question:

-in some species in which MLVA is used, some VNTRs occur in families, i.e. different VNTR loci share the same repeat unit. How will MLVAtype behave in such a case? It seems that all loci sharing an identical repeat motif will be (incorrectly) assigned the largest allele size.

**Answer:**

**All loci sharing an identical repeat motif will indeed be incorrectly assigned the largest allele size. For such species, an in silico PCR approach would be more appropriate. According to your recommendation (see your other later comment), we decided to focus the paper on *V. cholerae* where each repeat unit is only found in one locus.**

Question:
-conversely, tandem repeats are often not perfect. How will MLVAtype behave in such a case?

**Answer:**

**For such case, an *in silico* PCR approach would indeed be more appropriate. According to your recommendation (see your comment later), we have now restricted the focus of our application on *V. cholerae* where tandem repeats are perfect. It is worth noting that the problem of right-censoring applies only to a perfect repetition of the same motif. This also explains why right-censored data were not observed in the study of Vergnaud et al. Frontiers Microbiology 2018.**

Question:
-what is the rational for not taking into account contigs smaller than 2000 bp, especially given that in the present situation (Vibrio cholera VNTRs), the VNTR loci are shorter than 200 bp?

**Answer:**

**The 2000 bp threshold was modified to 1000 bp in order to be in line with literature. Small contigs are actually excluded because they are often non-informative [1, 2, 3 , 4] and associated with a low coverage.**

Question:
-Page 2 third $: the authors do not mention other published approaches for in silico MLVA typing. For instance Vergnaud et al. Frontiers Microbiology 2018 previously explored the possibility to deduce MLVA from WGS data was evaluated for Brucella using an in silico PCR approach.

**Answer:**

**We fully agree with the reviewer and thank him for his suggestion. This reference is now included in the reference list of the amended version of the paper.**

Question:
-Page 6, Table 4: please also provide the initial estimates before applying the “right-censorchip”. The authors seem to implicitly assume that Spades will never correctly reconstruct tandem repeat arrays longer than the k-mer size, but do not provide the data to show that.

**Answer:**

**We agree with the comment. In the amended version of the paper, this table is now provided as ‘supplementary file 1’ and commented in the “Results” section.**

Question:

-by default, Spades will explore multiple k-mer values (rather than a specified value). It would seem useful to provide in Table 4 the results obtained in these conditions.

**Answer:**

**We agree with the comment. In the amended version of the paper, these results are now provided in the ‘supplementary file 1’ and commented in the “Results” section.**

Question:
-if indeed Spades will not reconstruct tandem repeats longer than k-mer size, then why try to assemble reads when the read length is longer than available k-mer sizes (instead of recovering the reads of interest using tools such as Bbduk)?

**Answer:**

**One advantage of the current application is that you can run the MLVA typing on a shiny application very quickly and easily. This process (including data transfer and analyses) would be much longer if the MLVA profiles were directly typed from the reads. Therefore, deriving MLVA profiles directly from the reads was not tested in our study. As the current results showed no discrepancy between WGS data (after assembly) and Sanger sequencing data (gold standard), we focused the paper on deriving MLVA profiles from the assembly.**

Question:

-the in vitro MLVA assay does not seem to be correctly working yet, because the Bioanalyzer does not have a sufficient amplicon sizing resolution and/or because the allele calling (conversion from size estimate to repeat copy number) is not optimized, as indeed suggested by the authors in the first paragraph page 8. The error rates reported in Table 3 for the Bioanalyzer (above 50%!) and for GenScan (7 errors in 10 strains for the second locus) are not acceptable. What is the interest of backward compatibility of WGS with in vitro MLVA typing if the in vitro data is so bad, i.e. if MLVA does not seem applicable here? I believe that the Bioanalyzer (in)capacity to discriminate VNTR alleles with repeat units smaller than 8 bp has already been discussed in the literature, see for instance De Santis et al., BMC microbiology 2011. Regarding the GenScan errors, this is probably due to incorrect allele calling, resulting from slightly inexact size measurement by the capillary equipment (literature available, see for instance Hyytia-Trees et al., Foodborne Pathog Dis 2010). Once correctly set-up, there should be no errors at least when using the GenScan.

**Answer:**

**The objective of the paper was not to optimize Bioanalyzer-derived MLVA typing (albeit this can of course be done as previously reported by Lista et a. in the literature). Accordingly and to avoid any ambiguity about the imperfect Bioanalyzer results presented in the first version of the paper, this part was removed in the amended version.**

**Likewise, and as discussed supra, the purpose was to compare NGs results with existing published results. We did not really questioned the reliability of these published GenScan results. However, following the comment of the reviewers, we addressed this question to our foreign collaborators and they decide to review the initial GenScan-based MLVA profiles; a senior technician ran them again: all but one - unexplained - mismatches were corrected!**

**These errors in our first submission and the reviewer’s comment underpin therefore the value of the work of Hyytia-Trees et al concluding that “*proper training and experience is necessary to collect accurate information when using the GeneScan methodology”.***

**Albeit not 100% concordant, this concordance between Sanger- and GenScan-derived MLVA profiles is substantially improved in the amended version of the manuscript (Table 3, Figure 3).**

Question:
Table 5: include the cost estimate for the GeneScan method (where the assay can be run in a single multiplex PCR).
The estimated cost for PCR (10 € per PCR for reagent costs) seems a bit high.
Please clarify the indicated WGS cost: does this cover the making of the sequencing library? What read length?
More generally, Table 5 and the associated paragraph are poorly informative, it would be useful to try to estimate the overall cost, based upon commercial services prices.

**Answer:**

**The cost of the Bioanalyzer method was replaced by the cost of the GenScan method.**

**In addition, PCR costs were updated and a new reference was added to clarify the cost of WGS analysis.**

Question:
Page 7, paragraph on “Theoretical feasibility …” is not informative as is. Should either be developed, by being more specific, or deleted.

**Answer:
This paragraph was deleted, accordingly.**

Question:
Page 8, second paragraph, “the larger, the k-mer size, the better the accuracy of WGS-derived MLVA profiles.” The authors need to show the data (see previous remark on Table 4). Also please explain the reason for limiting the k-mer size to 175.

**Answer:**

**Page 8, second paragraph is based on Figure 3. This is specified in the amended version of the paper.**

**The reason for limiting the k-mer to 175 is justified as follows:**

**“*As previously reported, the length of repeat motifs should not exceed 174 nucleotides for V. cholerae, corresponding to 29 repetitions of a 6 nt motif [13]. Accordingly, the longer k-mer size (i.e. 175) proved to generate a correct MLVA profile with no censored data*.”**

Question:
Page 8, last paragraph before conclusion: the theoretical evaluation has not been done appropriately. The postulated 6 nt motif is not applicable to any of the MLVA assays commonly used in the given list of genus/species. Rather the design of the MLVAtype software indicates that it will have a very limited range. Indeed the MLVAtype web page at https://ucl-irec-ctma.shinyapps.io/NGS-MLVA-TYPING/ appears tailored for V. cholerae (and perhaps MLVA assays with up to five VNTR loci, and short and perfect repeat arrays). The article might be more convincing if focused on V. cholerae and its 10000 publicly available sequence reads archives.

**Answer:**

**We fully agree with the reviewer’s suggestion. The updated version of the paper focuses now only on *V.* *cholerae* MLVA application.**

Question:
-details,
Page 1 last $, Mycobacterium, Streptococcus etc are not species but genus. Please be more specific.

**Answer**

**Considering that the new version of the paper focuses on *V.* *cholerae,* and that the paragraph related to the theoretical feasibility has been removed in the new version of the paper, this sentence was removed.**

Question:
Page 2, first paragraph “Mounting evidence …” obviously and by definition Whole Genome Sequencing can only be better than the previous methods, no need to refer to “Mounting evidence”. The issue is cost, as detailed in the second paragraph.

**Answer**

**This sentence has been removed, as required.**

**References:**

1: Gurevich, Alexey, et al. "QUAST: quality assessment tool for genome assemblies." *Bioinformatics* 29.8 (2013): 1072-1075.

2: Bultman, Katherine M., et al. "Draft Genome Sequences of Type VI Secretion System-Encoding Vibrio fischeri Strains FQ-A001 and ES401." *Microbiology resource announcements* 8.20 (2019): e00385-19.

3: Rozanov, Aleksey S., et al. "Metagenome-Assembled Genome Sequence of Phormidium sp. Strain SL48-SHIP, Isolated from the Microbial Mat of Salt Lake Number 48 (Novosibirsk Region, Russia)." *Microbiology resource announcements* 8.31 (2019): e00651-19.

4: Parks, Dylan, et al. "Genome Sequence of Bacillus subtilis natto VK161, a Novel Strain That Produces Vitamin K2." *Microbiology resource announcements* 8.35 (2019): e00444-19.
