# Supplementary material for: Backward compatibility of whole genome sequencing data with MLVA typing using a new MLVAtype shiny application for Vibrio cholerae
Source: PLoS One. 2019 Dec 11;14(12):e0225848. doi: 10.1371/journal.pone.0225848 (PMC6905556; doi:10.1371/journal.pone.0225848)
Supplement: Attachment — Submitted filename: Response_to_reviewer.doc [file pone.0225848.s002.doc]

**We thank the Editor and the Reviewer for their careful reading of our manuscript. In revising our paper, we carefully followed the editor’s direction, and replied to the point by point questions of the reviewer.**

**We are confident that the answers provided and the corresponding modifications in the revised version will now meet the Editor and the Reviewer’s expectations.**

**We thank you in advance for your editorial work.**

**The authors**

Reviewer #1: The authors have significantly clarified and improved their report. A few points are written in a misleading way and can easily be improved.(unfortunately for the reviewer, lines are still not numbered)

Page 2, “However, it is worth noting that an in silico PCR approach to type MLVA in Brucella from WGS data was recently developed”

Would be more exact to indicate:
“However, it is worth noting that an in silico PCR approach to type MLVA from WGS data was recently developed and evaluated for Brucella”
Along this line of benchmarking, may be worth mentioning https://github.com/Papos92/MISTReSS

**Answer:**

**We fully agree with the reviewer and thank him for his suggestion that we integrated as such. The MISTReSS approach developed for salmonella was also cited.**

Page 3 second line “GeneScan determination was retrieved for Ugandan isolates from published
data [9]”
This sentence is misleading since it suggests that the chromatograms were published as part of the Bwire et al. 2018 publication. However what I understand is that the authors have in the course of the present investigation realized that the MLVA alleles calling published in the 2018 report was incorrect, and have reanalyzed the data. This is a different thing.

**Answer:**

**We fully agree with the reviewer. This point was clarified in the revised version of the manuscript.**

Page 3 second paragraph “method proposed by Kendall et al. [10], the formula had to be modified to better fit the sequence length of the motif and the position of the primers (Table 1). It is of note that, the original calculation formula was used for the VC0283 motif but with a modified reverse primer”

Not clear to me. Do the authors just mean that the use of the modified VC0283 reverse primer has no impact on the PCR product size? May be worth recalling the sequence of the previous primer, as in “(AGCCTCCTCAGAAGTTGAG instead of the previous XXXXX)”

**Answer:**

**This point was clarified in the revised version of the manuscript.**

“Locus” would be less ambiguous than “motif” (which refers to the repeat unit).

**Answer:**

**Motif was replaced by locus.**

Page 3, page 4 and elsewhere: two designations are used for loci VC0171 (alias VCA0171) and VC0283 (alias VCA0283), please harmonize

**Answer:**

**This was harmonized in the in the revised version of the manuscript**

Page 4: “returns the number of tandem repeats” and “increasing number (j=2, 3 .., k) of tandem repeats”:
may be clearer to replace “tandem repeats” by “tandemly repeated units”

**Answer:**

**We prefer to keep the original ‘number of tandem repeats’ in order to have a harmonized nomenclature with the other parts of the paper and literature (e.g. Variable number of tandem repeat [VNTR])**

Page 5 cost analysis paragraph, “Reagents costs for MLVA typing (5 motifs) of V. cholerae isolates were compared using three different methods (Table 5).”
The authors need to expand a little bit. Discuss the relative costs. Recall that Sanger sequencing was used only to produce a reference dataset, but not as a suggestion for routine MLVA typing (as shown, would make no sense in terms of cost).

**Answer:**

**We thank the reviewer for his suggestion that was implemented in the revised version of the manuscript.**

Table 5
Instead of “Motifs”, the authors probably mean “Loci”?

**Answer:**

**Yes, this was corrected.**

Cost estimates:
Sanger sequencing: are these commercial costs? Eight dollars for one PCR in terms of reagents and consumables seems a lot. Genscan-based typing: with only five loci, all five loci should be run in one multiplex PCR. Most laboratories running MLVA on Genscan-type of equipment and significant numbers of strains will multiplex the PCRs. Then the fair reagents cost estimate should be down to one PCR and one run per sample i.e. 11.4 USD.
WGS cost: indicating current best commercial prices (for sequencing 1 strain alone versus as part of a batch of 96) might be useful

**Answer:**

**If the five loci are amplified in a single multiplex PCR, then the cost for Genescan will be $11.4 US. We have indicated that this would be the cost if the five loci are amplified in a single PCR, but that at this time and at the best of our knowledge, such a pentaplex PCR has not been published. And the cost with published protocols is (2*8) for PCR plus (2*3.4) for fragment analysis since the amplified products are run as one duplex and one triplex. Thus, the total following published protocols is $22.8 US as indicated now in the table.**

Page 5, “However, it should be noted that a modification of either the primer” When running MLVA on a Genscan type of machine, the “formula” is useless. The allele calling software will call each allele base on its associated observed size range. This remark by the authors suggests that the authors are exporting raw size estimates from the Genscan, and then convert by allele calling using the “formula”. This is not the most recommended way to proceed, see available literature.

**Answer:**

**The Genescan software uses LIZ600 as an internal lane standard to determine the size in “basepairs” of the amplified fragments. The size of the fragments is reported as 148.05 basepairs as shown in the figure below copied from page 113 of the fragment analysis guide (**[**https://www.thermofisher.com/content/dam/LifeTech/global/Forms/PDF/fragment-analysis-chemistry-guide.pdf**](https://www.thermofisher.com/content/dam/LifeTech/global/Forms/PDF/fragment-analysis-chemistry-guide.pdf)**). The Genescan program can convert the size in basepairs to ordinal allele sizes using data from the scans if there are at least three different allele sizes. Whether the formula is computed by the Genescan program or the user, the starting basepair will still produce the same ordinal allele size. When the user does the conversions, there is a chance that errors made by the Genescan program in assigning the sizes of lane standard will be identified and a correction can be made. If the program does everything, we do not believe that this type of error will be identified and corrected. Further, as noted on page 107, the calling software does not work for repeats sizes larger than 7, as is the case for VC1650 where the repeat is 9 basepairs long.**

Page 5, “was applied in order to decrease the number of mismatches, especially those observed
with VC0437 and VCA0283.”
Quote Table 1 (and check locus names, see a previous remark)

**Answer:**

**This modification is implemented in the revised version of the manuscript.**

Page 7: “hence solving the well-recognized issue of backward compatibility with traditional MLVA typing methods.”
The software is not solving anything! There has never been an issue of backward compatibility when the sequencing reads are longer than the tandem repeat arrays. Recall that when tandem repeats contain internal variations, software for sequence assembly may be able to reconstruct correctly tandem repeats longer than the sequencing reads. Mention alternative approaches (the in silico PCR methods, including in terms of benchmarking https://github.com/Papos92/MISTReSS) and explain why the present approach is believed to be more appropriate at least for V. cholera MLVA.

**Answer:**

**The discussion was modified in order to discuss the** **complementarity of our approach with an *in silico* PCR method that we implemented in R for the purpose of this paper.**

I would suggest to merge Table 1 and S1 table as S1 table clearly illustrates the impact of the k-mer size.

**Answer:**

**Unfortunately, a fusion of both tables would make them too difficult to display and to read. Accordingly, we included separately this table (Table 5) as well as a table with the MLVA profiles obtained using the *in silico* PCR approach (Table 6) in this revised version of the manuscript.**

Discussion: the authors need to comment on the pros and cons of the approach they use here versus the more commonly used in silico PCR approach. In particular they need to indicate that the approach used here work in the Vibrio cholerae context because the VNTRs are unique (so the repeat unit sequence is locus-specific), the tandem repeat arrays are perfect, and there are no indels in the flanking sequences. This is an uncommon situation. They might indicate why they think this approach may be of interest when applicable. Do they think it is because it does not require to have assembled the flanking sequences?

**Answer:**

**We thank the reviewer for his suggestion. When the k-mer size is smaller than the repeated size, the contigs tend to be broken at the end of the repeated region. In that case, forward and reverse PCR primers are located on different contigs, making the *in silico* PCR approach inappropriate. Accordingly, and as suggested by the reviewer, the discussion was modified in order to discuss the** **complementarity of our approach with an *in silico* PCR method.**
